# Effectiveness of a Decision Aid Plus Standard Care in Surgical Management Among Patients With Early Breast Cancer: A Randomized Clinical Trial

**DOI:** 10.1001/jamanetworkopen.2023.35941

**Published:** 2023-10-02

**Authors:** Shalaka Joshi, Lakshmi Ramarajan, Naresh Ramarajan, Serenity S. Lee, Ojas Deshpande, Elizabeth Fernandes, Mitchelle Engineer, Gitika Srivastava, Vaibhav Vanmali, Sadhana Kannan, Rohini Hawaldar, Nita Nair, Vani Parmar, Purvi Thakkar, Garvit Chitkara, Sudeep Gupta, Rajendra Badwe

**Affiliations:** 1 Department of Surgical Oncology, Tata Memorial Centre, and Homi Bhabha National Institute, Mumbai, India; 2 Harvard Business School, Harvard University, Boston, Massachusetts; 3 Navya Network, Cambridge, Massachusetts; 4 Clinical Research Secretariat, Tata Memorial Centre and Homi Bhabha National Institute, Mumbai, India; 5 The Wharton School, University of Pennsylvania, Philadelphia; 6 Department of Medical Oncology, Tata Memorial Centre, and Homi Bhabha National Institute, Mumbai, India; 7 Homi Bhabha National Institute, Mumbai, India

## Abstract

**Importance:**

Patients with early breast cancer must choose between undergoing breast conservation surgery or mastectomy. This decision is often difficult as there are trade-offs between breast conservation and adverse effects, and women with higher decisional conflict have a harder time choosing the therapy that suits their preferences.

**Objective:**

To study the impact of a decision aid with a patient preference assessment tool for surgical decision-making on patients’ decisional conflict scale (DCS) score.

**Design, Setting, and Participants:**

This 3-group randomized clinical trial was conducted between June 2017 and December 2019 at a single high-volume tertiary care cancer center in Mumbai, India. A research questionnaire comprising 16 questions answered on a Likert scale (from 1, strongly agree, to 5, strongly disagree) was used to measure DCS scores and other secondary psychological variables, with higher scores indicating more decisional conflict. The Navya Patient Preference Tool (Navya-PPT) was developed as a survey-based presentation of evidence in an adaptive, conjoint analysis-based module for and trade-offs between cosmesis, adverse effects of radiotherapy, and cost of mandatory radiation following breast-conserving surgery. Adult patients with histologically proven early breast cancer (cT1-2, N0-1) who were eligible for breast-conserving surgery as per clinicoradiological assessment were included. Those who were pregnant or unable to read the research questionnaire or who had bilateral breast cancer were excluded. Data were analyzed from January to June 2020.

**Interventions:**

Patients were randomized 1:1:1 to study groups: standard care including clinical explanation about surgery (control), standard care plus the Navya-PPT provided to the patient alone (solo group), and standard care plus the Navya-PPT provided to the patient and a caregiver (joint group).

**Main Outcomes and Measures:**

The primary end point of the study was DCS score. The study was 80% powered with 2-sided α = .01 to detect an effect size of 0.25 measured by Cohen *d*, *F* test analysis of variance, and fixed effects.

**Results:**

A total of 245 female patients (median [range] age, 48 [23-76] years) were randomized (82 to control, 83 to the solo group, and 80 to the joint group). The median (range) pathological tumor size was 2.5 (0-6) cm. A total of 153 participants (62.4%) had pN0 disease, 185 (75.5%) were hormone receptor positive, 197 (80.4%) were human epidermal growth factor receptor 2 negative, 144 (58.6%) were of middle or lower socioeconomic status, and 114 (46.5%) had an education level lower than a college degree. DCS score was significantly reduced in the solo group compared with control (1.34 vs 1.66, respectively; Cohen *d*, 0.50; SD, 0.31; *P* < .001) and the joint group compared with control (1.31 vs 1.66, respectively; Cohen *d*, 0.54; SD, 0.31; *P* < .001).

**Conclusions and Relevance:**

The results of this study demonstrated lower decisional conflict as measured by DCS score following use of the online, self-administered Navya-PPT among patients with early breast cancer choosing between breast-conserving surgery vs mastectomy.

**Trial Registration:**

Clinical Trials Registry of India Identifier: CTRI/2017/11/010480

## Key Points

**Question:**

Does a decision-making aid have an effect on decisional conflict scale score among women diagnosed with early breast cancer choosing between breast conservation surgery and mastectomy?

**Findings:**

This randomized clinical trial including 245 women found that women who used the decision aid with patient preference assessment tool had a statistically improved decisional conflict scale score compared to those who did not.

**Meaning:**

The findings in this study suggest that decision aids and patient assessment tools may provide valuable information for treatment decision-making in patients with early breast cancer.

## Teaser

This randomized clinical trial evaluates the effect of a decision-making aid and patient preference assessment tool in surgical decision-making among patients with early-stage breast cancer.

## Introduction

Surgical treatment for early breast cancer involves either mastectomy or breast conservation surgery and radiation.^1,2,3^ Both the treatments are oncologically equal. The decision between mastectomy and breast-conserving surgery is personal, and patients have different attitudes and perceptions that inform their decision. The cost and adverse effects of radiotherapy is a deterrent for some. Patients with lower education status, higher number of consultations with surgeons or health care professionals, and those in minoritized racial or ethnic groups tend to favor mastectomy.^4,5,6,7^ Potential advantages of breast-conserving surgery are better body image, a sense of sexual well-being, and preservation of femininity.^8,9^ Shared decision-making is advisable in this setting.

The rate of breast-conserving surgery is particularly low in lower middle–income countries due to lack of awareness of safety of breast-conserving surgery, socioeconomic disparities, concerns about additional expenditures, perceived fears of radiation, lack of radiotherapy units, and an element of overall neglect toward women’s health and quality of life.^10,11,12^ Decision aids are interventions designed to help patients make choices by providing information about options and outcomes relevant to a person’s health status.^13,14^ They have been validated in randomized clinical trials and found acceptable, useful, and desirable by patients and their physicians for various health-related conditions.^15,16^ The impact of a decision aid can be assessed by reduction in decisional conflict or a person’s state of uncertainty about a course of action. The decisional conflict scale (DCS) is a widely used evaluation measure in studies of decision aid use among patients.^17^ When converted to a 100-point scale, with every 1 unit decrease in DCS, people are 59 times less likely to change their mind.^15,18^

Conventional decision aids rely on administration by clinicians, thereby limiting access, and do not measure the impact of patient factors, such as preference for autonomy, traditional gender roles, and caregiving responsibilities.^4,7,19^ Conjoint analysis, an established research method, has been used in shared decision-making research to understand how patients value different attributes of treatment. The current study relies on a decision aid that uses an adaptive conjoint methodology to assess patient preference, in which subsequent questions are modified based on participants’ replies to the prior questions. This is known to better represent a real-world way of decision-making and to have a better impact for participants.^20^ Furthermore, in the Asian setting, nationally representative studies have found that shared decision-making can be influenced by key family members, often without adequate participation by the female patients themselves.^19,21^

We designed the Navya–Patient Preference Tool (Navya-PPT), a self-administered, online, adaptive, conjoint analysis-based decision aid and patient preference assessment tool. We hypothesized that using the Navya-PPT would reduce participants’ decisional conflict. We conducted a 3-group randomized clinical trial to measure the impact of the Navya-PPT on DCS score. In the Indian socioeconomic scenario, women often do not make decisions on their own but are influenced by a primary male family member’s opinion.^19,21^ For this reason, we assessed the efficacy of the intervention when provided to patients alone and when provided to patients and a caregiver in order to provide cultural context for our results.

## Methods

The study was approved by the institutional ethics committee of Tata Memorial Centre and registered on CTRI. The study protocol is in Supplement 1. All participants provided written informed consent. The study followed the Consolidated Standards of Reporting Trials (CONSORT) reporting guideline.

### Study Population

Patients at Tata Memorial Centre, a high-volume tertiary care cancer center in Mumbai, India, who had a histologically proven diagnosis of early breast cancer (cT1-2/cN0), were included. Patients younger than 18 years and those who had bilateral breast cancer, were pregnant, or were unable to read or comprehend a research questionnaire were excluded. All patients had a thorough triple assessment for suspicion of cancer, including clinical examination, imaging evaluation (digital mammography and ultrasonography when necessary), and needle biopsy. Eligibility for breast-conserving surgery was assessed by the Breast Cancer Disease Management Group at the Tata Memorial Centre (comprising surgeons, radiologist, radiation, and medical oncologist). Patients and their relative(s) were then counseled in the outpatient clinic by a surgeon regarding the suitability and preference for their surgical choices (breast-conserving surgery vs mastectomy). Neither breast-conserving surgery nor mastectomy was represented as favorable in the discussion. Relevant queries from patients were answered to help them understand the nuances of each surgical procedure, and the point of mandatory radiation following breast-conserving surgery was emphasized. Eligible patients were then screened to participate in the study. Operating surgeons were not aware of the patients’ responses to the questionnaire.

The Navya–Patient Preference Tool (Navya-PPT) (eFigure in Supplement 2) is an intervention jointly developed by researchers at Tata Memorial Center, Navya Network, and Harvard University. This adaptive, conjoint analysis-based module represents trade-offs between 3 main attributes: ability to preserve cosmesis with breast-conserving surgery, adverse effects, and additional cost of mandatory radiation following breast-conserving surgery. The conjoint analysis survey was created using Sawtooth Software version 8.4.8 (Sawtooth Software) and communicated evidence supporting each treatment option in a pictorial format. Patients entered their preferences for each attribute on a Likert scale from 1 to 9. This determined the patients’ values for each of the trade-offs represented by survey questions. Sawtooth Software version 8.4.8 was used to compute the overall score for each treatment preference. The Navya-PPT and research questionnaire were also translated from English into Hindi and Marathi, and patients could use the questionnaire in their language of choice.

The research questionnaire was used to collect patient demographic characteristics, study end points, and patient preferences for surgery. The primary end point was DCS score. The eFigure in Supplement 2 provides a snapshot of the 16 questions used in the research questionnaire to obtain DCS score. Secondary end points were other psychological scales measuring shared decision-making (ie, Autonomy Preference Index-Decision Making [API-DM],^22^ Traditional-Egalitarian Gender Roles [TEGR],^23^ and a caregiving role scale formulated for the Indian context), selected based on the Indian context. More details about the research questionnaire are available in eMethods in Supplement 2. The research questionnaire also recorded the patient’s preference for type of surgery (recorded in the research questionnaire as lumpectomy with radiation, mastectomy, or unsure) with respect to the actual surgery received. Only the research questionnaire was administered to patients in the control group whereas research questionnaire was administered after the Navya-PPT in both the intervention groups.

### Randomization

Patients were centrally block randomized (sequence generated using varying block sizes) with allocation concealment (1:1:1) into 3 groups using a between-participant design. All patients and their relatives received information regarding their surgical choice in the outpatient clinic as standard care and were then randomized. The first group (control) consistent of standard care, in which patients received clinician counseling on surgical options and filled out the research questionnaire. In the second group (the solo group), patients received standard care plus the Navya-PPT followed by the research questionnaire, which they filled out alone (ie, without a key family member). In the third group (the joint group), patients received standard care and the Navya-PPT followed by the research questionnaire, which they filled out in the presence of a key family member. Almost 90% of the key family members were male.

### Study End Points

The primary end point of the study was change in DCS score among women undergoing surgery for primary operable breast cancer after administering the Navya-PPT. The 16-item research questionnaire measured DCS score using a Likert scale from 1 (strongly agree) to 5 (strongly disagree), with higher scores indicating more decisional conflict. A mean score was calculated for each patient.

The secondary end points of the study the effectiveness of the interventions on clinical outcomes, such as breast-conserving surgery rates, the concordance of Navya-PPT–reported preference with the final surgery received by the patient, and the effect of Navya-PPT on DCS score stratified by other psychological indices. Additional psychological scales, including API-DM, TEGR, and caregiving, were also measured on a 1 to 5 Likert scale. The internal reliability of the psychological scales were measured by Cronbach α.^24^ We also calculated correlations (Spearman and Pearson ρ) between these psychological scales with known demographic variables as a measure of external validity.

### Statistical Analysis

A Cohen effect size of 0.2 to 0.5 has been validated in published literature as meaningful difference between control and intervention.^25^ With a power of 80% and a 2-sided α of .10 (to allow for comparisons between control and each intervention group using Bonferroni correction), to detect an effect size of 0.25 (Cohen *d*) in DCS score with randomization into 3 groups, the sample size would need to include a total of 228 patients (*F* test analysis of variance; fixed effects). Assuming a drop-out rate of 10%, we included 85 patients in each group for a total of 255 patients (G-POWER).^26,27^ Stratification criteria used were age (age 50 years and younger vs older than 50 years), socioeconomic status as calculated by the Kuppuswamy Index (≥16 vs <16), and educational level (some college or lower vs college graduation and above).^28^

Patient demographic, clinicopathologic, and disease characteristics were reported as numbers and percentages. We used a significance threshold of .05 in all analyses. Univariate analysis was performed using Pearson χ^2^ or Fisher exact test. We used 1-way analysis of variance to compare the mean DCS scores of the 3 study groups, followed with *t* tests for pairwise comparisons between study groups, and we report Cohen *d* for effect size.

The match proportion between the patient’s stated preference with the actual surgery received (coded as match if lumpectomy was preferred and received or mastectomy preferred and received; coded as mismatch otherwise) was calculated to understand whether preference match was higher in any 1 of the 3 groups. We also ran univariate and multivariate regression analyses to study the effect of Navya-PPT on DCS score stratified by other psychological indices. All analyses were conducted through SPSS version 25 (IBM). Data were analyzed from January to June 2020.

## Results

The study randomized 255 women between June 2017 and December 2019. Ten patients were excluded from analysis after randomization (Figure).^29^ The results are reported on the 245 patients as per protocol analysis.

**Figure.**
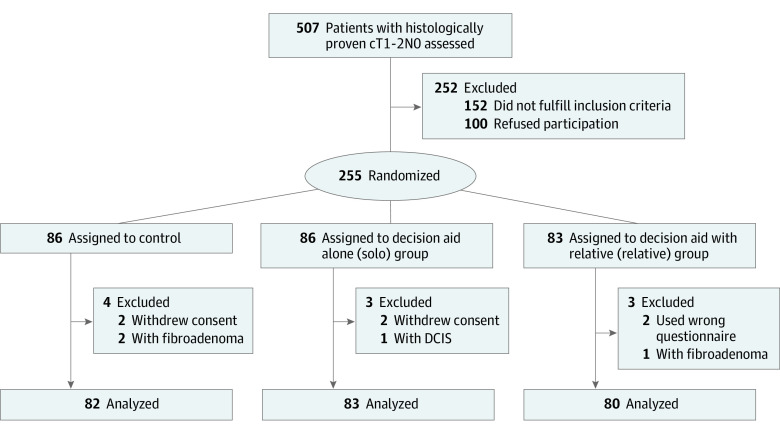
CONSORT Diagram DCIS indicates ductal carcinoma in situ.

The median (range) age of the cohort was 48 (23-76) years, and 137 of 245 participants (55.9%) were premenopausal or perimenopausal. The median (range) pathological tumor (pT) size was 2.5 (0-6) cm with 79 participants (32.2%) having pT less than or equal to 2 cm and 156 (63.7%) having pT greater than 2 cm. Axillary lymph node status on histopathology report was negative in 153 participants (62.4%), hormone receptor (estrogen receptor/progesterone receptor) status was positive in 185 (75.5%), and human epidermal growth factor receptor-2 was negative in 197 (80.4%). Of note, 144 participants (58.6%) were of lower or middle socioeconomic status and 114 (46.4%) had not completed college. Further details of the clinicopathological characteristics are provided in Table 1.

**Table 1.** Demographic and Disease Characteristics and Stratification Criteria of 245 Randomized Patients

Variable	No. (%)				*P* value
	Total (N = 245)	Control group (n = 82)	Solo group (n = 83)^a^	Joint group (n = 80)^b^	
Age, median (range), y	48 (23-76)	48 (23-72)	48 (28-70)	50 (26-76)	NA
Menopausal status					
Pre/peri	137 (55.9)	46 (56.1)	46 (55.4)	45 (56.2)	.99
Post	108 (44.1)	36 (43.9)	37 (44.6)	35 (43.8)	
Histological grade (MRB)					
Low	41 (16.7)	10 (12.2)	17 (20.5)	14 (17.5)	.41
High	203 (82.8)	71 (86.5)	66 (79.5)	66 (82.5)	
Unknown^c^	1 (0.4)	1 (1.2)	NA	NA	
pT, cm					
Mean (median; range)	2.5 (2.5; 0-6)	2.6 (2.5; 0-5.3)	2.4 (2.4; 0-5.4)	2.5 (2.4; 0.5-6)	.78
≤2	73 (29.8)	24 (29.3)	24 (28.9)	25 (31.3)	
2-5	156 (63.7)	54 (65.9)	55 (66.3)	47 (58.8)	
>5	3 (1.2)	1 (1.2)	1 (1.2)	1 (1.3)	
Unknown^d^	13 (5.3)	3 (3.7)	3 (3.6)	7 (8.8)	
Total axillary nodes dissected, mean (median; range)	12.2 (10.0; 0-40)	11.8 (9.0; 0-38)	12.5 (11.0; 2-40)	12.2 (11.0; 2-38)	.40
Pathological axillary nodal status					
0	153 (62.4)	49 (59.7)	55 (66.3)	49 (61.2)	.40
1-3	66 (26.9)	23 (28.0)	23 (27.4)	20 (25.0)	
4-10	9 (3.7)	6 (7.3)	1 (1.2)	2 (2.5)	
>10	4 (1.6)	1 (1.2)	1 (1.2)	2 (2.5)	
Unknown^d^	13 (5.4)	3 (3.6)	3 (3.6)	7 (8.7)	
Hormone receptor status (ER/PR)					
Positive	185 (75.5)	59 (72.0)	65 (78.3)	61 (76.3)	.61
Negative	59 (24.1)	22 (26.8)	18 (21.7)	19 (23.7)	
Unknown^c^	1 (0.4)	1 (1.2)	NA	NA	
*ERBB2* ^e^					
Negative	197 (80.4)	68 (82.9)	65 (78.3)	64 (80.0)	.76
Positive	46 (18.8)	13 (15.9)	18 (21.7)	15 (18.8)	
Unknown	2 (0.8)	1 (1.2)	NA	1 (1.3)	
Type of surgery					
BCS	202 (82.4)	67 (81.7)	71 (85.6)	64 (80.0)	.49
Mastectomy	30 (12.3)	12 (14.6)	9 (10.8)	9 (11.2)	
Unknown^d^	13 (5.3)	3 (3.7)	3 (3.6)	7 (8.8)	
Language of research questionnaire					
English	83 (33.9)	21 (25.6)	33 (39.8)	29 (36.3)	.25
Hindi	98 (40.0)	40 (48.8)	28 (33.7)	30 (37.5)	
Marathi	64 (26.1)	21 (25.6)	22 (26.5)	21 (26.2)	
**Stratification criteria**					
Age, y					
≤50	151 (61.6)	51 (62.2)	51 (61.4)	49 (61.3)	NA
>50	94 (38.4)	31 (37.8)	32 (38.6)	31 (38.7)	
Educational status					
≤College degree	114 (46.5)	38 (46.3)	38 (45.8)	38 (47.5)	NA
≥College degree	131 (53.5%)	44 (53.7)	45 (54.2)	42 (52.5)	
Kuppuswamy index score					
≤16	144 (58.6)	48 (58.5)	48 (57.8)	48 (60.0)	NA
>16	101 (41.3)	34 (41.5)	35 (42.2)	32 (40.0)	

Abbreviations: BCS, breast conservation surgery; ER, estrogen receptor; MRB, Modified Richardson Bloom for histological grade; NA, not applicable; PR, progesterone receptor; pT, pathological tumor size.

^a^ The solo intervention provided study materials to participants only.

^b^ The joint intervention provided study materials to participants and a caregiver.

^c^ One patient had excision biopsy elsewhere, and grade and receptor status were unknown.

^d^ Thirteen patients did not undergo surgery at our center and surgical details were not available.

^e^ Two patients did not have a fluorescence in situ hybridization test to confirm *ERBB2 *status.

### Primary End Point: DCS Score

The mean (SD; range) DCS score for the full study population was 1.43 (0.61; 1.00-4.81). Table 2 shows the difference in DCS score by treatment group (*F*_2,242_ = 8.84; *P* < .001). DCS score was significantly reduced in the solo group compared with the control group (1.34 vs 1.66, respectively; *t*_163_ = 3.19; 95% CI, 0.12-0.52; *P* < .001; Cohen *d, *0.50; SD, 0.31), as well as in in the joint group compared with the control group (1.31 vs1.66, respectively; *t*_160_ = 3.42; 95% CI, 0.15-0.55; *P* < .001 Cohen *d,* 0.54; SD, 0.31).

**Table 2.** Effect of Decision Aid on Decisional Conflict Scale (DCS)

Study group	DCS, mean (SD; 95% CI)	Mean difference (95% CI)	Cohen *d* (SD) (compared to control)	*P* value
Control	1.66 (0.79; 1.48 to 1.83)	NA	NA	NA
Solo^a^	1.34 (0.44; 1.24 to 1.43)	0.32 (0.12 to 0.52)	0.50 (0.31)	<.001
Joint^b^	1.31 (0.46; 1.20 to 1.41)	0.35 (0.15 to 0.55)	0.54 (0.31)	<.001
Solo and joint combined	1.32 (0.45; 1.25 to 1.39)	0.33 (−0.49 to −0.18)	0.59 (0.29)	<.001

Abbreviation: NA, not applicable.

^a^ The solo intervention provided study materials to participants only.

^b^ The joint intervention provided study materials to participants and a caregiver.

### Secondary End Points

Out of 245 patients, surgical details were available for 232 patients who underwent surgery at our institution. Analysis for the primary end point of DCS score was carried out for the 245 patients who completed the protocol. The overall rate of breast-conserving surgery was 82.4% (202 of 232 [87.1%] when applied only to those for whom surgical details were available) and was equal across the 3 groups. Of the 245 patients, 83 (33.9%) took the survey in English, 98 (39.9%) in Hindi, and 64 (26.2%) in Marathi. Most patients (196 of 245 [80%]) indicated strong agreement on a 1 (strongly agree) to 5 (strongly disagree) Likert scale item stating that “the survey questions were easy to understand” (mean [SD], 1.27 [0.68]).

### Effect of Navya-PPT on DCS Score Stratified by Other Demographic and Psychological Indices

We saw no significant differential effect of Navya-PPT on DCS score when stratified for TEGR and API-DM scores (high vs low). The interaction of caregiving role scale (high vs low) and Navya-PPT was not significant (*F*_1,241_ = 3.73; *P* = .055 [analysis of variance]) (Table 3). To further test the effect of Navya-PPT on DCS score stratified for other demographic and disease variables, experimental conditions (Navya-PPT with and without family member) and psychological variables (API-DM, TEGR, and caregiver role), we ran univariate and multivariate ordinary least squares regressions using standardized variables. eTable 1 in Supplement 2 shows the regression coefficients of each factor. None of the demographic factors (ie, age, education status, and socioeconomic status), pT size, or psychological factors (as reflected by API-DM, TEGR, and caregiver role scores) significantly impacted DCS.

**Table 3.** Differences in Decisional Conflict Scale (DCS) Within Subgroups^a^

Subgroup	Differences in DCS by experimental group			
	Control vs solo^b^	*P* value	Control vs joint group^c^	*P* value
Low API-DM	*t*_84_ = 2.85	.01	*t*_81_ = 2.45	.01
High API-DM	*t*_77_ = 1.86	.06	*t*_77_ = 2.73	.01
Low TEGR	*t*_60_ = 1.49	.07	*t*_65_ = 2.08	.06
High TEGR	*t*_101_ = 3.25	.01	*t*_93_ = 3.12	.001
Low caregiving	*t*_23_ = 0.08	.47	*t*_26_ = −0.23	.41
High caregiving	*t*_132_ = 3.75	.001	*t*_202_ = 4.57	.001

Abbreviations: API-DM, Autonomy Preference Index-Decision Making; TEGR, traditional-egalitarian gender roles.

^a^ API-DM, TEGR, and embeddedness were stratified into low and high groups using a median split. We report 1-sided *P* values in independent sample *t* test, as we expected the control group to have higher DCS than the other groups where the decision aid was used.

^b^ The solo intervention provided study materials to participants only.

^c^ The joint intervention provided study materials to participants and a caregiver.

### Preference-Surgery Match

Of the 242 patients who expressed a preference for type of surgery, 169 (70%) said they wanted lumpectomy, 37 (15%) said they wanted mastectomy, and 36 (15%) said they were unsure. Overall, 202 patients received breast-conserving surgery, and 30 received mastectomy, leading to a match percentage of 57.9% in the control group, 71.3% in the solo group, and 75.3% in the joint group (Table 4). However, a χ^2^ analysis of patients’ preferences (overall χ^2^, 6.67; *P* = .15) indicated that a greater proportion of patients reported being unsure of their preference in the control group (18 of 79 [22.8%]) compared to those in the solo group (8 of 83 [9.6%]), a significant difference at the *P* < .05 level. This result is consistent with the higher decisional conflict scores in the control group. Although the joint group (10 of 80 [12.5%]) had similar results to the solo group, the proportion of unsure patients in the control group and the joint group were not significantly different from each other. We found that those who experienced surgery that matched their preference also reported significantly lower DCS score compared with those with a mismatch (mean [SD], 1.32 [0.48] vs 1.71 [0.79]; *F*_1,227_ = 22.12; *P* < .001) (eAppendix 1 in Supplement 2).

**Table 4.** Patient Preference, Actual Surgery Received, and Preference/Surgery Match

	No. (%)			
	Control	Solo group	Joint group	Total
Patient preference				
BCS	52 (65.8)	61 (73.5)	56 (70.0)	169 (69.8)
Mastectomy	9 (11.4)	14 (16.9)	14 (17.5)	37 (15.3)
Unsure	18 (22.8)	8 (9.6)	10 (12.5)	36 (14.9)
Total	79 (100.0)	83 (100.0)	80 (100.0)	242 (100.0)
Type of surgery received				
BCS	67 (84.8)	71 (88.8)	64 (87.7)	202 (87.1)
Mastectomy	12 (15.2)	9 (11.3)	9 (12.3)	30 (12.9)
Total	79 (100.0)	80 (100.0)	73 (100.0)	232 (100.0)
Patient preference vs type of surgery				
No match	32 (42.1)	23 (28.8)	18 (24.7)	73 (31.9)
Match	44 (57.9)	57 (71.3)	55 (75.3)	156 (68.1)
Total	76 (100.0)	80 (100.0)	73 (100.0)	229 (100.0)

Abbreviation: BCS, breast conservation surgery.

### Internal Reliability and External Validation of the Research Questionnaire

The Cronbach α values for DCS score, API-DM, TEGR, and caregiver role were 0.94, 0.74, 0.76, and 0.66 respectively, suggesting good internal reliability. Also, correlations in the data set were consistent with prior research and trends expected in the real world, suggesting external validity as well (eTable 2 in Supplement 2). Specifically, higher patient educational level was negatively associated with API-DM (higher API scores indicate less desire for decision-making) (*r*, −0.19; *P* < .001), as was higher socioeconomic status (*r*, −0.23; *P* < .001). Higher TEGR scores (more traditional gender roles) were associated with less education (*r*, −0.38; *P* < .001), lower socioeconomic status (*r*, −0.31; *P* < .001), lower education of the patient’s husband (*r*, −0.26; *P* < .001), and having more children (*r*, 0.21; *P* < .001). Caregiver role was associated with lower participant education (*r*, −0.15; *P* < .001) and lower socioeconomic status (*r*, −0.17; *P* < .001) (eAppendix 3 in Supplement 2).

## Discussion

To our knowledge, this randomized clinical trial represents the first report of the use of a decision aid to choose between breast-conserving surgery and mastectomy in the Indian population, using a self-administered, multilingual, online, interactive decision tool based on adaptive conjoint analysis to assess patient preference. We found a significant improvement in DCS score through the use of the Navya-PPT. Similar findings were reported in a randomized clinical trial from Canada with improvement of DCS score (1.40 vs 1.62; difference, 0.22; SE, 0.10; *P* = .02) following administration of a decision aid among patients with early breast cancer.^30^ A recent systematic review^14^ of 7 studies of patients with early breast cancer facing surgical choice determined improvement in psychological changes and quality of life by reducing DCS score using a decision aid, albeit reiterating the need for better decision aids. Our study found reduced DCS scores using the Navya-PPT in a lower middle-income country with a population mostly of lower socioeconomic and educational status. We developed an intervention that was simple and could easily be self-administered. Such self-administered interventions outside the medical encounter have previously been shown to increase patient involvement and access to shared decision-making.^31,32^ The use of an adaptive conjoint analysis methodology to assess preference and translation into 2 common vernacular languages are other unique features of the Navya-PPT that make it easy to use and may have contributed to its benefit.

Further, the median age of women in our study was 48 years and 55% were premenopausal or perimenopausal, representing a younger population, consistent with the country’s population pyramid.^33^ With a median tumor size of 2.5 cm, 62% node negativity, and 75% hormone receptor positivity, the cohort in our study reflects a favorable prognostic subgroup of patients with breast cancer with good long-term disease-related outcomes. This has important implications for long-term quality of life with breast-conserving surgery. The overall breast-conserving surgery rate was 82.4%, which is higher than that reported in prior national literature for similar patient populations.^34,35^ This relatively high rate of breast-conserving surgery may be due to location, as upwards of 60% of early breast cancer at our institute undergo breast-conserving surgery.^36^ However, higher patient involvement due to the study itself might have contributed to increasing the rate of breast-conserving surgery in all 3 groups. The counseling of patients regarding surgical choices was done in the outpatient clinic by surgical members of the Breast Cancer Disease Management Group. The surgeons were a mix of both sexes and followed a uniform counseling policy and vernacular language that suited the patient.

We believe the reduction of DCS score in the intervention groups and the increased match of preference with final surgery type were a result of fewer patients being unsure of their choice after using the decision aid. In lower middle-income countries such as India, decisions between breast-conserving surgery and mastectomy are often determined paternalistically,^37^ either by the patients’ treating physicians or through the influence of key family members, who often are male. This can hinder autonomy in decision-making. In our study, the presence of key family members varied naturally in the control group that provided standard care. In the intervention groups, we planned for the use of the decision aid with and without family members present and observed reduced DCS scores compared to control in both conditions. This suggests the decision aid can help lower DCS score both with and without family members present. Patient preference and surgery match were significantly higher in both the intervention groups compared to the control group. This supports the use of the intervention as promoting value-concordant decision, further improving the strength of the intervention.

Another unique feature of our study was the inclusion of additional psychological indices, such as API-DM, TEGR, and caregiver role scales. Women who are less autonomous, more traditional, and more responsible for caregiving functions in the family likely have inherent disparities in access to shared decision-making. We show that the use of Navya-PPT can reduce DCS score in women regardless of decreased autonomy, adherence to more traditional gender norms, or higher caregiving burden. This bodes favorably for engaging women in shared decision-making with an appropriately designed decision aid.

### Strengths and Limitations

The strengths of our study are that it is, to our knowledge, the first large, randomized study conducted in a lower middle-income setting to study the impact of a novel intervention incorporating a decision aid that can be administered simply, without strain on medical resources, with the use of vernacular languages to facilitate patient understanding. We found reduced decisional conflict about the choice between breast-conserving surgery and mastectomy across socioeconomic and educational strata. The findings suggest that the Navya-PPT can be used to help patients with early breast cancer in this important decision-making process.

The study also has limitations. Previous studies have shown counseling by female surgeons to influence decision in favor of breast-conserving surgery.^38^ However, we did not document surgeon sex in standard care counseling or the sex and involvement of other family members before accrual in the study. The effect of these extraneous factors should be equally distributed across the 3 groups due to the randomized nature of the study. A few other limitations of our study include lack of measurement of DCS score prerandomization and the noninclusion of specific quality-of-life parameters to document ultimate benefit of the intervention, which we endeavor to achieve in the future.

## Conclusions

In this randomized clinical trial, an online, self-administered, adaptive intervention—Navya-PPT—used outside of the clinician encounter reduced decisional conflict as measured by DCS score and provided an avenue to shared decision-making. The findings indicate that the Navya-PPT may help patients with early breast cancer make the important decision between breast-conserving surgery and mastectomy.

## Notes

Supplement 1.
Trial Protocol

Supplement 2. **eFigure.** Snapshot of the Navya-PPT a) pictorial depiction of breast conservation surgery and mastectomy as a part of the decision aid, b) snapshot of conjoint analysis tool, and c) Research Questionnaire (RQ)- subscale- 16-point questions related to the primary end point, Decisional Conflict Scale (DCS) **eTable 1.** Univariable and Multivariable Regression Analysis of Prediction of DCS **eTable 2.** Correlations of variables in the Research Questionnaire **eTable 3.** Means and Standard Deviations of Secondary Endpoints by Experimental Arm **eAppendix 1.** Additional Psychological Scales assessed in the Research Questionnaire – API-DM, TEGR and CG **eAppendix 2.** Validation of Psychological Scales in the RQ **eAppendix 3.** Preference surgery match

Supplement 3.
Data Sharing Statement
