# Supplementary material for: Effectiveness of a Decision Aid Plus Standard Care in Surgical Management Among Patients With Early Breast Cancer: A Randomized Clinical Trial
Source: JAMA Netw Open. 2023 Oct 2;6(10):e2335941. doi: 10.1001/jamanetworkopen.2023.35941 (PMC10546236; doi:10.1001/jamanetworkopen.2023.35941)
Supplement: Supplement 1. — Trial Protocol [file jamanetwopen-e2335941-s001.pdf]

A Randomized Controlled Trial of Navya Patient Preference Tool- An Online Self-Administered Adaptive Conjoint Analysis Based Decision Aid, for women undergoing surgery for early breast cancer

PROTOCOL

**Protocol Version**	:	**01**
**Protocol Ver. Date**	:	**October 2015**

Investigators

**Principal Investigator**	:	Dr. Rajendra A. Badwe
**Co-Principal Investigators**	:	Dr. Shalaka Joshi
		Dr. Abhay K Kattepur
**Co-Investigators**	:	Dr. Vani Parmar
		Dr. Nita S. Nair
		Dr. Sudeep Gupta
		Prof. Lakshmi Ramarajan, PhD*
		Mrs. Sadhana Kannan
		Mr. Vaibhav J. Vanmali

Collaborators

**Navya Network**	:	Dr. Naresh Ramarajan
		Ms. Gitika Srivastava
***Harvard Business School**	:	Prof. Lakshmi Ramarajan, PhD

Background

There are two surgical management strategies for patients with early, operable breast cancer; breast conservation therapy (which includes breast conservation surgery and radiotherapy) and modified radical mastectomy. Both of these strategies are clinically equivalent in terms of overall survival of patients.[1,2,3]However, these strategies differ from one another in toxicity and cost/convenience for the patient in undertaking the treatment and a perceived element of fear of recurrence. The best option for a patient will depend on how she values the different treatment

attributes associated with each of the two management strategies. Therefore, either of the two options - Breast Conservation Therapy or Mastectomy could be superior personal preference for a given patient. The potential advantages of breast conservation are better body image, a sense of sexual well-being, less body consciousness, a greater sense of femininity, and psychological comfort. On the other hand, there have been reports of post mastectomy depression, anxiety, shame, and occasional suicidal thoughts following mastectomy.[4] Other than that there is an often perceived fear of cancer recurrence in the same breast after breast conservation, a common reason for patients demanding mastectomy even for early breast cancer.

There is a large variation in the pattern of surgical treatment for breast cancer mainly due to lack of involvement or participation of the patients in the decision making process. In the study by *Katz et al*[5], there was an increase in mastectomy rates (33%) when the patient involvement in the decision making process increased. Discussion on recurrence of disease and effects of radiation tilted the balance towards mastectomy. In the study by *Bleicheret al*[6], a total of 1259 patients with non-invasive or localized invasive breast cancer were randomized into mastectomy or breast conservation groups and the role of patient in decision making was studied. The factors which proved to be significantly favoring mastectomy were active patient involvement in decision making process, lower education level, number of surgeons or health care providers consulted and non-white race.

Decision aids are "interventions designed to help people make specific and deliberative choices among options by providing information about the options and outcomes that is relevant to a person's health status". They are designed to transfer unbiased, complete, and accessible information to the patient and enable her participation in decision making[7]. Over time, decision aids have been validated in randomized trials and found acceptable, useful, and desirable by patients and their physicians. About 40% of patients refer to internet based decision aids before meeting their physician.[8] It has been shown that decision aids are effective in improving patient knowledge, patient participation in decision making, and decision quality.[9]The decisional

conflict index (DCI) is designed to measure the patient's comfort with the decision making process in terms of how well-informed they feel, the clarity of their values, how supported they feel in the DM process and their level of uncertainty. It ranges from 1 to 5. The effectiveness of a decision aid has been tested in various fields of medicine as well as oncology, in at least 34 randomized controlled trials, majority of them pertaining to breast and prostate cancer. In most of these studies, the decisional conflict was reduced moderately.

There are three significant limitations to the current design of decision aids. First, they are primarily designed to be used during the clinical encounter, and to be administered by a physician, which is resource intensive. Second, these decision aids use methods that compare treatments to each other on similar attributes (such as decision boards, or standard gamble technique) and ask patients to choose a treatment based on their consideration of the attributes. The limitation with this approach is that it only passively transfers knowledge of attributes such as cost and quality of life, but asks the patient to holistically choose a treatment after consideration of these attributes. This places the onus of the decision back on the patient who is struggling with the choice in the first place.

In a particular study by Brace et al [10], the awareness of the physicians towards Decision aids was studied and found that 69% of the respondents were aware of such aids but only 24% of them actually utilized them.

Alternative methods such as conjoint analysis based techniques have been studied in the medical context.[11,12] Conjoint analysis emphasizes the choice of attributes themselves, and asks the patient to make risk/benefit tradeoffs such as efficacy vs. side effects in an adaptive, recursive way, until the patient's underlying values are clarified. These values are mapped to the treatment choice, leading to a more obvious and intuitive decision making process. This is similar to the way clinician's approach decision making with a patient. Currently, there are no digital, web based, interactive, conjoint analysis base decision aids available for patients with breast cancer that will actually drive them towards a decision.

Finally, decision aids have largely been studied in the context of the patient alone, devoid of family input or other key decision makers. For example, surgical decisions in breast cancer are heavily influenced by family roles, gender roles, autonomy and self-image. These factors heavily influence a patient's decision making, and are not accounted for in traditional patient focused decision aids. All of the above limitations significantly reduce the possibility of delivering effective decision aids to patients at a large scale.

Patient preference is an important consideration in clinical decision making, especially when there is clinical equipoise. One frequent scenario in which patient preference matters is when women diagnosed with breast cancer are confronted with the choice between breast conservation therapy and mastectomy. Traditionally, decision aids are designed to be administered by physicians, which is inefficient. Further, decision aids force the patient to pick from a menu of treatment options instead of assessing the intrinsic values driving the decision. Navya bridges this gap by developing an online, self-administered, conjoint analysis based, interactive decision tool that assesses risk/benefit tradeoffs of the underlying decision criteria like efficacy versus toxicity. The proposed randomized control trial aims to demonstrate reduction in decisional conflict and improved clinical outcomes by using the Navya Patient Preference Tool as compared to usual care.

We hypothesize that gender roles and autonomy play a significant role in a woman's ability to make her own medical decisions. A secondary aim of the trial is to determine whether the optimal use of a decision aid includes participation by both the woman and her key family member. The proposed trial randomizes the use of the conjoint analysis decision tool to a woman alone or in conjunction with her key family member (who in most Indian families may be a male family member such as husband, father or brother).

A Randomized Controlled Trial of Navya Patient Preference Tool- An Online Self-Administered Adaptive Conjoint Analysis Based Decision Aid, for women undergoing surgery for early breast cancer

PROTOCOL

The Navya Patient Preference Tool (Navya PPT)

The Navya Patient Preference Tool, (Navya PPT) jointly developed in an endeavor between Tata Memorial Center, Navya Network, and Harvard Business School, conveys the evidence behind each of the management strategies or treatment options, and helps the informed patient assess their preferences for each of the attributes (such as side effects, cost and convenience and the cosmetic aspect of keeping the breast) by using a conjoint analysis technique. It offers an interactive, digital, online interface such that the decision making process can happen without requiring excessive time involvement from the physician. Yet, it is designed to be at the level of quality that is necessary for the patient to understand her treatment options, assess her decision making criteria (attributes), be guided on what her underlying decision making values or risk preferences are, and ultimately lead to a treatment decision that makes her feel comfortable, reassured, and empowered. Finally, the proposed trial of the Navya Patient Preference Tool (PPT) is designed to study the influence of family role, gender role, and autonomy in decision making to better inform who would be the best consumer of this decision aid (patient vs. patient and key family member).In the Indian context, for decisions in breast cancer, the woman patient and her key family member (who is likely to be a male family member such as husband, father or son) are studied.

Hypothesis

1. The online survey i.e Navya PPT (patient preference tool) (decision making aid) will reduce the “decisional conflict index” (DCI) of patients diagnosed with early breast cancer while choosing between mastectomy and breast conservation.

2. Participation of a woman’s self-identified key family member will reduce decisional conflict, especially in women highly embedded in the family, with traditional gender identity or limited sense of autonomy.

A Randomized Controlled Trial of Navya Patient Preference Tool- An Online Self-Administered Adaptive Conjoint Analysis Based Decision Aid, for women undergoing surgery for early breast cancer

PROTOCOL

Objectives

The proposed randomized control trial will study the use of the Navya Patient Preference Tool (Navya PPT) in the above mentioned scenario in which women with early breast cancer are confronted with the equivocal choice of Breast Conservation Therapy (BCT) or Mastectomy. In this scenario, patient preference matters; and therefore the effectiveness of the Navya Patient Preference Tool (Navya PPT) will be studied. This trial will test the following two hypotheses:

Primary objective

To study the change in Decisional Conflict Index (DCI) of women undergoing surgery for primary, operable breast cancer after administering a decision making tool which is the Navya Patient Preference Tool (PPT)

Secondary objectives

1) To study its effect on clinical outcomes such as patient satisfaction rate, breast conservation rate

2) To study the concordance with Navya's PPT, concordance with physician's recommendation, and follow through of decision by patient

3) To study the effect of Navya PPT on clinical outcome and DCI stratified by their Autonomy Preference Index (API), by Traditional Egalitarian Gender Role (TEGR) and by Family embeddedness

Exhibit- A version of the Navya Patient Preference Tool is attached which includes,

1) **Online Conjoint survey**- a version of the questions presented in a survey format by the Sawtooth Software that uses the conjoint analysis technique to determine the survey takers values for each of the attributes represented by the survey questions

2) **Research questionnaire**- the questions to determine a patient's autonomy and her and her key family member's behavioral attributes and influences in decision making

The survey as well as the Research questionnaire has been translated in Hindi and Marathi, which are also attached.

Methodology

The proposed trial is a randomized controlled trial. Patients will be randomized into 3 arms.

1) **Arm 1**- Control arm- Usual care- where the patient will be explained pertinent points regarding her choices of breast conservation and mastectomy for the surgical management of her breast cancer, by the treating doctors.

2) **Arm 2**- Experimental arm1- women will be asked to take the online conjoint analysis based decision aid (which is the Navya PPT) alone.

3) **Arm 3**- Experimental arm 2- women will take the online conjoint analysis based decision aid with a self-identified key family member.

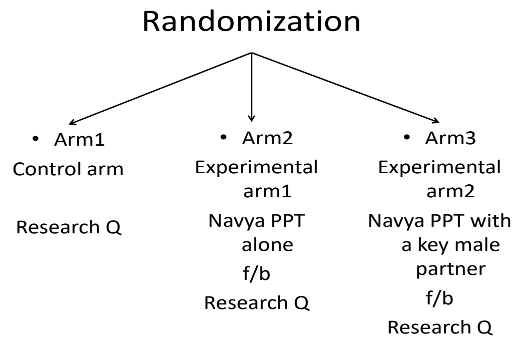

We have conducted a pilot study where 15-20 individuals (health care professionals and patients) have answered the survey and they were able to understand the questionnaire. Also to validate the internal consistencies, an analysis will be carried out at the end of 50 patients being recruited.

All women in all three arms will be given a research questionnaire to calculate their "decisional conflict index" and understand other aspects such as "autonomy preference index" or "traditional gender role".

Eligibility

Inclusion Criteria

1. Women with histologically proven breast cancer (by core biopsy/FNAC/excision biopsy)
2. Upfront operable cT1,T2 N0 breast cancer
3. Eligible for Breast Conservation (absence of multicentricity, or extensive microcalcifications in mammogram), as deemed at surgical planning evaluation of the patient after complete diagnostic data is gathered
4. Written, informed consent

Exclusion criteria

1. Patients under 18 of years of age
2. Patients who are pregnant
3. Patients who cannot comprehend the questionnaire due to psychiatric illness or inability to understand the study consent

Sample Size and Power Estimates

A moderate Cohen's effect size of 0.2-0.5 has been validated in published literature as a meaningful difference between control and intervention group. [11]

With a power of 80% and a 2-sided alpha of 0.01 (to allow for correction for multiple comparisons), to detect a difference on 0.25 (effect size, Cohen's d) in the DCI, with randomization into 3 arms, the sample size would be a total of 228 patients (F test- ANOVA, fixed effects). To compensate for loss of data and patients, we would include 85 patients in each arm making a total of 255 patients. Randomization will be done centrally using block-randomization.

Patients will be randomly assigned to either control arm- usual care (only the research questionnaire will be administered to calculate the DCI) or experimental arm 1- Navya PPT on their own or experimental arm 2- Navya PPT with a self-identified key family member, using stratified block randomization with varying block sizes (two and four). Stratification factors will include patients' socio-economic status which will be calculated using the Kuppuswamy index (16 and above versus 15 and below), age (<50 versus above 50) and educational qualification (<=12th, graduation & above).

Data Analysis and Outcome Measures

Outcome Variables: The primary outcome is the Decisional Conflict Index (DCI).Secondary outcomes are Patient Satisfaction Scale, Rate of BCT Vs Mastectomy, Concordance with Navya Patient Preference Tool, Concordance with Physician, Follow through with Decision (Compliance).

Study/Independent Variables: Demographic Screen, Conjoint Analysis (utilities and importance of the patient's preference), Autonomy Preference Index (API), Gender Role Index (Traditional Egalitarian Gender Role (TEGR), Appearance Scale, Care-giving Scale, Resiliency Scale)

Sawtooth Software uses Adaptive Conjoint Analysis methodology to measure/rank the importance of each attribute (Side Effects, Breast Retention, Local Recurrence, Cost/ Convenience of Treatment) in decision making. This is interpreted as patient's preference for BCT or Mastectomy.

Gender Role Index will be analyzed using a multivariate linear regression model to assess relationships between Gender Role, Intervention (Woman vs Joint) and Decisional Conflict.

References

1. Fisher B, Anderson S, Bryant J, Margolese RG, Deutsch M, Fisher ER, et al. Twenty-year follow-up of a randomized trial comparing total mastectomy, lumpectomy and lumpectomy plus irradiation for the treatment of invasive breast cancer. N Engl J Med 2002;347:1233-41.

2. Poggi MM, Danforth DN, Sciuto LC, Smith SL, Steinberg SM, Liewehr DJ, et al. Eighteen-year results in the treatment of early breast carcinoma with mastectomy versus breast conservation therapy: The National Cancer Institute randomized trial. Cancer 2003;98(4):697-702.

3. Litière S, Werutsky G, Fentiman IS, Rutgers E, Christiaens MR, Limbergen EV, et al. Breast conserving therapy versus mastectomy for stage I–II breast cancer: 20 year follow-up of the EORTC 10801 phase 3 randomised trial. Lancet Oncol 2012;13:412-19.

4. Stavrou D, Weissman O, Polyniki A, Papageorgiou N, Haik J, Farber N, et al. Quality of life after breast cancer surgery with or without reconstruction. Open Access Journal of Plastic Surgery 2009;9:161-168.

5. Katz SJ, Lantz PM, Janz NK, Fagerlin A, Schwartz K, Liu L, et al. Patient involvement in surgery treatment decisions for breast cancer. J Clin Oncol 2005;23:5526-5533.

6. Bleicher RJ, Abrahamse P, Hawley ST, Katz SJ, Morrow M. The influence of age on the breast surgery decision making process. Ann Surg Oncol 2008;15(3):854-862.

7. O'Connor AM, Stacey D, Entwistle V, et al: Decision aids for people facing health treatment or screening decisions. Cochrane Database of Systematic Reviews. CD001431, 2003 DOI: 10.1002/14651858.CD001431

8. Vordermark D. Patient information and Decision Aids in oncology: Need for communication between patients and physician. J Clin Oncol 2010;28(29):e567.

9. O'Brien MA, Whelan TJ, Villasis-Keever M, Gafni A, Charles C, Roberts R, et al. Are cancer-related decision aids effective? A systematic review and meta-analysis. J Clin Oncol 2009;27:974-985.

10. Brace C, Schmoeker S, Huang H, Victor C, McLeod R, Kennedy ED. Physician awareness and attitudes toward Decision Aids for patients with cancer. J Clin Oncol 2010;28(13):2286-2292.

11. Pieterse AH, Stiggelbout AM, Marijnen CAM. Methodologic evaluation of adaptive conjoint analysis to assess patient preferences: an application in oncology. Health expectations 2010;13:392-405.

12. Kievit W, van Hulst L, van Riel P, Fraenkel L. Factors that influence Rheumatologists' decision to escalate care in RA: results from a choice based conjoint analysis. Arthritis Case Res 2010;62(6):842-847.

Kuppuswamy socio-economic status scale calculation

For ease of stratification, we have clubbed <16 (middle/lower middle, lower/upper lower and lower) and =/>16 (upper, upper middle) as 2 categories. Please find the table provided below for the reference.

(A) Education Score		
1	Profession or Honours	7
2	Graduate or post graduate	6
3	Intermediate or post high school diploma	5
4	High school certificate	4
5	Middle school certificate	3
6	Primary school certificate	2
7	Illiterate	1

(B) Occupation Score		
1	Profession	10
2	Semi-Profession	6
3	Clerical, Shop-owner, Farmer	5
4	Skilled worker	4
5	Semi-skilled worker	3
6	Unskilled worker	2
7	Unemployed	1

(C) Monthly family income in Rs		Score	Modified for 1998 [3] in Rs	Modified for 2012 in Rs
1	≥ 2000	12	≥ 13500	≥32050
2	1000-1999	10	6750 - 13499	16020 – 32049
3	750-999	6	5050 - 6749	12020 – 16019
4	500-749	4	3375 - 5049	8010 – 12019
5	300-499	3	2025 - 3374	4810 – 8009
6	101-299	2	676 - 2024	1601 – 4809
7	≤ 100	1	≤ 675	≤ 1600

Total Score	Socioeconomic class
26-29	Upper (I)
16-25	Upper Middle (II)
11-15	Middle/Lower middle (III)
5-10	Lower/Upper lower (IV)
<5	Lower (V)

264
