# Supplementary material for: Effectiveness of a Decision Aid Plus Standard Care in Surgical Management Among Patients With Early Breast Cancer: A Randomized Clinical Trial
Source: JAMA Netw Open. 2023 Oct 2;6(10):e2335941. doi: 10.1001/jamanetworkopen.2023.35941 (PMC10546236; doi:10.1001/jamanetworkopen.2023.35941)
Supplement: Supplement 2. — eFigure. Snapshot of the Navya-PPT a) pictorial depiction of breast conservation surgery and mastectomy as a part of the decision aid, b) snapshot of conjoint analysis tool, and c) Research Questionnaire (RQ)- subscale- 16-point questions related to the primary end point, Decisional Conflict Scale (DCS) eTable 1. Univariable and Multivariable Regression Analysis of Prediction of DCS eTable 2. Correlations of variables in the Research Questionnaire eTable 3. Means and Standard Deviations of Secondary Endpoints by Experimental Arm eAppendix 1. Additional Psychological Scales assessed in the Research Questionnaire – API-DM, TEGR and CG eAppendix 2. Validation of Psychological Scales in the RQ eAppendix 3. Preference surgery match [file jamanetwopen-e2335941-s002.pdf]

Supplemental Online Content

Joshi S, Ramarajan L, Ramarajan N, et al. Effectiveness of a decision aid plus standard care in surgical decision-making among patients with early breast cancer: a randomized clinical trial. *JAMA Netw Open*. 2023;6(1):e2335941. doi:10.1001/jamanetworkopen.2023.35941

eFigure. Snapshot of the Navya-PPT a) pictorial depiction of breast conservation surgery and mastectomy as a part of the decision aid, b) snapshot of conjoint analysis tool, and c) Research Questionnaire (RQ)- subscale- 16-point questions related to the primary end point, Decisional Conflict Scale (DCS)

eTable 1. Univariable and Multivariable Regression Analysis of Prediction of DCS

eTable 2. Correlations of variables in the Research Questionnaire

eTable 3. Means and Standard Deviations of Secondary Endpoints by Experimental Arm

eAppendix 1. Additional Psychological Scales assessed in the Research Questionnaire – API-DM, TEGR and CG

eAppendix 2. Validation of Psychological Scales in the RQ

eAppendix 3. Preference surgery match

This supplemental material has been provided by the authors to give readers additional information about their work.

© 2023 Joshi S et al. *JAMA Network Open.*

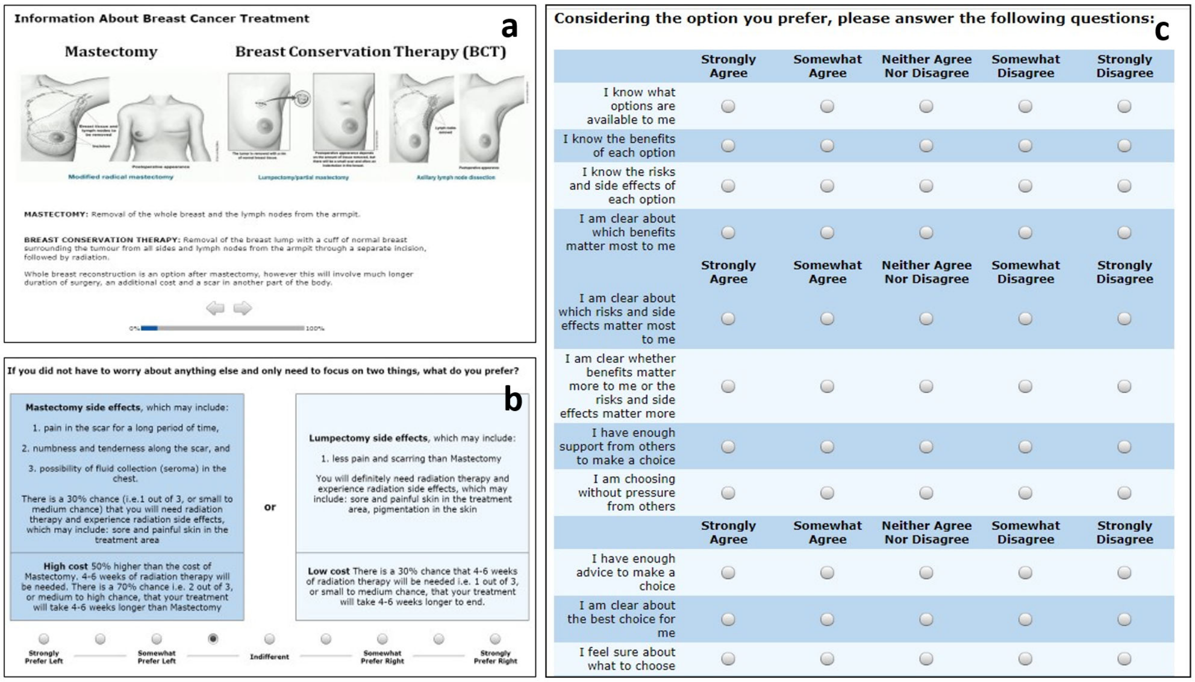

eFigure. Materials

Snapshot of the Navya-PPT a) pictorial depiction of breast conservation surgery and mastectomy as a part of the decision aid, b) snapshot of conjoint analysis tool, and c) Research Questionnaire (RQ)- subscale- 16-point questions related to the primary end point, Decisional Conflict Scale (DCS)

© 2023 Joshi S et al. *JAMA Network Open.*

eTable 1. Univariable and Multivariable Regression Analysis of Prediction of DCS

	**Univariable**	**Multivariable**
	Coefficient (St. Error)	Coefficient (St. Error)
**Solo (Arm 2)**	-.24[a] (0.13)	-.57[c] (0.16)
**Joint (Arm 3)**	-.31[b] (0.14)	-.66[c] (0.16)
**Age (>50 years)**	-.06 (0.13)	.00 (0.13)
**Education (>12th class)**	-.05 (0.13)	-.21 (0.18)
**KI (≥ 16)**	-.00 (0.13)	.06 (0.18)
**API-DM**	-.09 (0.06)	-.06 (0.07)
**TEGR**	-.07 (0.06)	-.10 (0.08)
**CG**	-.13 (0.06)	-.10 (0.07)
**PT Size**	.16 (0.14)	.15 (0.14)

Solo/Arm 2 and Joint/Arm 3 were measured as dummy variables; the base category is the Control/Arm 1 condition. Age, Education, KI, and PT Size were measured as binary variables.

[a] $p < 0.10$, [b] $p < 0.01$, [c] $p < 0.001$.

© 2023 Joshi S et al. *JAMA Network Open.*

eTable 2. Correlations of variables in the Research Questionnaire

	M	SD	1	2	3	4	5	6	7	8	9	10
**1. Patient's Age [% >50 years]**	.38	.49	—									
**2. Patient's Education [% > 12th]**	.54	.50	-.04	—								
**3. KI [% ≥ 16]**	.41	.49	.06	.67[a]	—							
**4. PT Size [% > 2]**	.69	.47	-.04	-.02	-.13	—						
**5. Husband's Education**	1.63	.48	.11	.50[a]	.38[a]	.02	—					
**6. Number of Children**	1.96	.92	.19[a]	-.26[a]	-.20[a]	.07	-.11	—				
**7. DCS**	1.43	.61	-.07	.11	.10	-.03	-.03	-.06	**α = .94**			
**8. API-DM**	4.38	.78	-.03	-.19[a]	-.23[a]	.05	-.10	.10	-.09	**α = .73**		
**9. TEGR**	3.45	1.18	.02	-.38[a]	-.31[a]	.06	-.26[a]	.21[a]	-.07	.37[a]	**α = .76**	

© 2023 Joshi S et al. *JAMA Network Open.*

**10. CG**	4.42	.81	-.01	-.15[b]	-.17[a]	.04	-.11	.11	-.13[b]	.26[a]	.27[a]	**α = .66**

[a] p < .01, [b] p <0.05 Pairwise correlations with binary variables (Variables 1 to 5) report Spearman's rho; all other pairings report Pearson's rho.

Directionality of scales: API-DM—higher is less desire for autonomy; TEGR—higher is more traditional beliefs about gender roles; CG—higher is greater commitment to caregiving responsibilities.

eTable 3. Means and Standard Deviations of Secondary Endpoints by Experimental Arm

**Variable**	**Control (Arm 1)**		**Solo (Arm 2)**		**Joint (Arm 3)**		**Total**	
	**M**	**SD**	**M**	**SD**	**M**	**SD**	**M**	**SD**
**API-DM**	4.48	0.75	4.28	0.84	4.39	0.75	4.38	0.78
**TEGR**	4.03	0.79	3.79	0.81	3.73	0.99	3.85	0.87
**CG**	4.41	0.78	4.47	0.73	4.37	0.91	4.42	0.81
**Preference/Surgery Match**	0.58	0.50	0.71	0.46	0.75	0.43	0.68	0.47

© 2023 Joshi S et al. *JAMA Network Open.*

eAppendix 1. Additional Psychological Scales assessed in the Research Questionnaire – API-DM, TEGR and CG

Examples of API-DM indicating less desire for autonomy were 6 items with higher scores; a sample item being 'your doctor should make important decisions and not you', TEGR – 6 items with higher scores indicating more traditional belief; a sample item being 'Ultimately, woman should submit to her husband's decision'; and CG – 4 items with high scores indicating stronger commitment to caregiving responsibilities; a sample item being 'I believe it is my duty as a wife to take care of my mother-in-law'. All items were measured on a 5-point Likert scale, where 1 = 'Strongly disagree' and 5 = 'Strongly agree, with mean score being calculated for each scale.

eAppendix 2. Validation of Psychological Scales in the RQ

Quality of a scale is traditionally evaluated in terms of validity; the extent to which an instrument measures what it claims to measure, rather than something else and reliability; the extent to which an instrument can be expected to give the same measured outcome when measurements are repeated(38). We measured several constructs and examined their associations. We found a high internal reliability of the psychological scales in the study. Importantly, results suggest that DCS was correlated with other variables in theoretically expected ways, providing some external validation as well.

Internal reliability of all scales was appropriate, as measured with Cronbach's alpha: Autonomy Preference Index—Decision-Making (API-DM) subscale (α = 0.73), Traditional-Egalitarian Gender Role (TEGR) (α = 0.76), and Caregiving (CG) (α = 0.66). Correlations in the dataset were consistent with prior research and trends expected in the real world. As shown in **eTable 2**, higher educational level of the patient was negatively associated with API-DM (higher is less desire for decision-making) (r=-0.19, $p<0.01$), as was higher SES (r=-0.23, $p<0.001$),

© 2023 Joshi S et al. *JAMA Network Open.*

suggesting more desire for involvement in DM. Higher TEGR scores (more traditional) were associated with less education (r=-0.38, p<0.01), lower SES (r=-0.31, p<0.01), less education of the patient's husband (r=-0.26, p<0.01) and having more children (r=0.21, p<0.01). CG was associated with less education of the participant (r=-0.15, p<0.05) and lower SES (r=-0.17, p<0.01).

Examining the correlations between the scales, TEGR (higher is more traditional) is significantly associated with API-DM (higher is less desire for decision-making) (r=0.37, p<0.01), suggesting more traditional beliefs about gender roles are associated with less desire for participation in DM. Greater CG were also positively associated with API-DM (r=0.26, p<0.01) and TEGR (r=0.27, p<0.001), suggesting less desire for involvement in DM and more traditional gender role beliefs. DCS was negatively correlated with CG (r=-0.13, p<0.05), and was not significantly correlated with API-DM nor TEGR. There was no significant interaction between TEGR and treatment arm. CG had a marginally significant interaction with Arm 2 (Solo) (B = -.22, p = .06). As a robustness check of experimental condition on DCS, we conducted ANCOVAs to control for other variables. In an ANCOVA with both demographic and scale variables ($F(10, 199) = 2.92, p < .01$), treatment arm remains a significant predictor of DCS ($F(2,199) = 9.17, p < .001$).

We also examined an exploratory hypothesis specifically about API-DM, to examine whether patients with lower autonomy preference may have experienced lower decisional conflict when in the joint condition and found a marginally significant interaction with Arm 3 (Joint) in predicting DCS ($B = -.21, p = .09$). These findings suggest potential role of SDM (Arm 3 conditions) in patients with lower preference for autonomy.

© 2023 Joshi S et al. *JAMA Network Open.*

eAppendix 3. Preference surgery match

Of the 242 patients who expressed their preferences for type of surgery, 70% patients said they want lumpectomy, 15% said they want mastectomy, and 15% said they were unsure (**eTable 3**). Overall, 202 patients received BCS, and 30 patients received MRM. However, a chi square analysis of patient's preferences (overall $X^2(4,N=242) = 6.67$, $p = .15$) indicated that a greater proportion of patients reported being unsure of their preference in Arm 1 (Control) (22.8%) compared to those in Arm 2 (Solo) (9.6%), a difference significant at the $p<0.05$ level. This result is consistent with the higher decisional conflict scores in Arm 1 (Control). Though Arm 3 (Joint) (12.5%) trended similarly as Arm 2 (Solo), the proportion of unsure patients in Arm 1 (Control) and Arm 3 (Joint) were not significantly different from each other.

Furthermore, 82.4% (± 6%) of patients underwent surgery of choice as determined by Navya-PPT. As shown in **eTable 3**, we conducted a chi square analysis to determine if proportions of preference/surgery match differed between the three conditions ($X^2(2, N=229)=5.77$, $p=0.056$); results indicated that patients in Arm 3 (Joint) were more likely to receive surgery that matched their preference (75%) than patients in Arm 1 (Control) (58%). Patients in Arm 2 (Solo) followed a similar pattern (71% match in Arm 2 vs. 58% match in Arm 1), although this was not significantly different at the 5% level.

We further conducted exploratory analyses to understand the relationship between the experimental conditions, DCS and matching of surgery with patient's preferences. We found that those who experienced surgery that matched their preference also reported significantly lower DCS ($M_{match} = 1.32 < M_{mismatch} = 1.71$, $F(1,227) = 22.12$, $p<0.001$).

© 2023 Joshi S et al. *JAMA Network Open.*
