# Supplementary material for: Effectiveness of a Decision Aid Plus Standard Care in Surgical Management Among Patients With Early Breast Cancer: A Randomized Clinical Trial
Source: JAMA Netw Open. 2023 Oct 2;6(10):e2335941. doi: 10.1001/jamanetworkopen.2023.35941 (PMC10546236; doi:10.1001/jamanetworkopen.2023.35941)
Supplement: Supplement 3. — Data Sharing Statement [file jamanetwopen-e2335941-s003.pdf]

Data Sharing Statement

Joshi. Effectiveness of a Decision Aid Plus Standard Care in Surgical Management Among Patients With Early Breast Cancer. *JAMA Netw Open*. Published October 02, 2023. doi:10.1001/jamanetworkopen.2023.35941

Data
Data available: Yes
Data types: Deidentified participant data
How to access data: PI email ID
When available: With publication

Supporting Documents
Document types: Other (please specify)
Additional Information: Original protocol
How to access documents: Uploaded with submission
When available: With publication

Additional Information
Who can access the data: Researchers
Types of analyses: Meta-analysis, systematic review
Mechanisms of data availability: After signed data access agreement
